# Five times a quarter of a century at Instituto Oswaldo Cruz

**DOI:** 10.1590/0074-02760250006

**Published:** 2026-05-08

**Authors:** Adeilton Alves Brandão

**Affiliations:** 1 Fundação Oswaldo Cruz-Fiocruz, Instituto Oswaldo Cruz, Laboratório de Biologia Molecular e Doenças Endêmicas, Rio de Janeiro, RJ, Brasil

**Keywords:** public health, anniversary, innovation, infectious disease

## Abstract

The pathway of Instituto Oswaldo Cruz (IOC) runs parallel with the astonishing series of events in the 20th century, which might be viewed as a dispute between hope and ideological belief.

As a centennial organisation the IOC has witnessed the same set of events and perhaps experienced some of them, but in most cases, it has been unable to influence or deter them.

Since its beginnings the IOC has been engaged in addressing public health challenges and either by serendipitous findings or by the scientific inquiry it has brought some *Res Novae* and ignited sparks of hope that one day most of the infectious diseases that proliferate in Brazil might be controlled.

In this perspective essay to introduce the series of articles in celebration of the 125th Anniversary of the IOC I will sketch an overview of its journey in the context of some 20th century events through a quarter of a century time frame.

The 20th century was once thought to be “short”,^1^ and at its final decade the “end of history” was declared.^2^ In reality, it was neither short nor it saw the end of history. The 20th century was full of innovations and revolutions (political, social, economic, scientific, technological) but it also witnessed mass destruction, extreme violence, prejudice, persecutions, hunger, poverty and the emergence (or re-emergence) of devastating infectious diseases. At the final count, the 20th century was not so different from the past ones except for the scale and intensity of the events mentioned above. Most of these events were precisely the results of actions caused by the hands and mind of *Homo sapiens*. Above all, the 20th century was an ultimate dispute between hope and ideological belief. On one hand the hope that science, technology, economic development, global trade, free speech and liberal democracy would give us peace and prosperity. On the other hand, the belief that collectivist ideologies, imposed on people through violent methods and guided by ruthless totalitarian leaders, would correct all of the mistakes human beings have made since birth. The goal was to bring the paradise of abundance, equality and happiness back to us, but the result was dreadful: millions of human lives lost!

Despite the drawbacks in the first quarter of the 21st century hope is still enduring and motivating us to go forward. Nonetheless the charmers of dreadful nightmares are at the next corner, waiting for an opportunity to capture the attention and support of those who are poor, hungry and desperate (any resemblance to a PhD candidate *status* might not be a mere coincidence).

The pathway of Instituto Oswaldo Cruz (IOC) runs parallel with the astonishing series of events in the 20th century. As a centennial organisation it has witnessed the same events and perhaps experienced some of them, but in most cases, it has been unable to influence or deter them. They occurred regardless of what the working people at the Institute have been doing in these five quarters of a century. Most but not all events. A little bit of influence has certainly been exerted by tireless researchers in and around the Moorish building in the lands close to coastal swamps at the outskirts of Rio de Janeiro city, then the capital of the newly born Brazilian Republic.

The IOC displayed at its beginnings the Latin expression *pro salute populi* and either by serendipitous findings or by the scientific inquiry and reasoning it has brought some *Res Novae* and ignited sparks of hope that one day most of the infectious diseases that proliferate in Brazil might be controlled.

In this perspective essay to introduce the series of articles in celebration of the 125th Anniversary of the IOC I will sketch an overview of its journey in the context of some 20th century events through a quarter of a century time frame.

**First quarter of a century**

“*...he who knows neither fatigue nor discouragement and dedicates long hours to active work, entirely oblivious to the pleasures of the outside world, living from science and by science*.”

(Carlos Chagas, in celebration of Adolpho Lutz 70th birthday)^3^

The first 25 years of the 20th century was remarkable as prelude of what would come in the next years, *e. g.*, bloody and massively destructive world wars (First World War, 1914-1918), influenza pandemic (1918) and colonialist disputes that redrawn frontiers and re-shaped the African continent and the southeast Asia. These events were somewhat connected with the working field of the Instituto Soroterápico Federal, an organisation for infectious diseases and public health research created in May 1900, and later renamed Instituto Oswaldo Cruz (Federal Decree 6,891 March 19th, 1908). This connection might also be grasped by the initiative to rename “colonial medicine” into a new brand, tropical medicine, that happened in the XV International Congress of Medicine in Lisbon, Portugal, 1906. The arising of “tropical medicine” was not only the result of the typical specialisation that follows the increase of human knowledge and accumulation of scientific evidence. In a certain way, it served the purpose and efforts of the European powers to maximise the extraction of resources from African and Asian tropical colonies.^4^ The brutal colonial efforts were to some extent hampered by the presence in these regions of viruses, bacteria, protozoa, worms and the respective insect vectors, that in their struggle for survival (or in a reductionism view, the act of living to transmit their genes to offsprings, *i. e.*, gene propagation) might cause serious trouble for anyone who crossed their path. The biological diversity in these regions was both an opportunity for colonial resources exploitation and a threat to those who venture into it. Altogether these facts contributed to the creation of organisations exclusively dedicated to research and training in tropical medicine, *e. g.* the majority of European tropical medicine institutes. It is worth mentioning that some of these tropical medicine institutes have publicly made a *mea culpa* about their role in contributing to colonial extractivism and exploitation.

As a former colony of Portugal that got its independence in 1822 Brazil was not indifferent to these events, though no longer being directly exploited by the colonial powers. With lands located in the tropics Brazil was severely affected by most of the infectious diseases described so far, a scenario of neglect and abandonment that was compounded by the 300 plus years of forced labour by enslaved Africans. It is in this context of brute force geopolitics, colonialism, early globalisation, social inequalities and public health challenges that the IOC unveiled (somewhat serendipitously) one of the most important discoveries in the human infectious diseases research in the 20th century: the Chagas disease or American Trypanosomiasis.^5^ As is common to the reports of completely *Res Novae* the announcement of a new trypanosomiasis was received with scepticism and harsh debates by some influential members of the Brazilian medical community.^6^ ^,^ ^7^ Carlos Chagas, the researcher who described the new disease, had to wait at least two decades to get broad recognition thanks to the efforts of an Argentinian researcher, Salvador Mazza, who got similar results working in Argentina in the 1920-30 decade.^8^ ^,^ ^9^

Amidst the concerns and hard work about diagnostics, control and surveillance of the human infectious diseases in Brazil during its first 25 years, there was another job to be done by researchers at IOC: to venture into deep Brazil, the poorest and most remote regions of the country. In this period, many expeditions were launched towards places such as the *sertões* of the northern east, the southern regions and the lands at the frontier of Bolivia, Paraguay, Argentina and Uruguay. More than a hundred years later, the notes from researchers leading these expeditions are surprisingly updated with respect to the challenges for biodiversity research, conservation of the natural landscape, the human occupation and land use, and most importantly, the precarious health and social conditions of their inhabitants. Here is the list of the expedition’s reports from the first quarter of a century published in Memórias do Instituto Oswaldo Cruz.^10^ ^,^ ^11^ ^,^ ^12^ ^,^ ^13^

**Second quarter of a century**

“*...the demonstration, through his achievements in Manguinhos, of the importance of science as an essential element for the progress and culture of a nation, awakening, with this example, an increasingly widespread interest in scientific research in the most diverse sectors of Brazilian activities.*”

(Henrique Aragão, at the 50th Anniversary of IOC)^14^

In Brazilian political history the year of 1937 represents the consolidation of a fascist-like dictatorship by Getulio Vargas (1930-1945), aiming to build a “new state”, strengthen the nation and guarantee a future of “order and progress” to the country. It was expected that governmental organisations such as IOC would help to pave the way leading to a high standard of life for the Brazilian population. Effective treatment of bacterial infections by new antibiotics was coming to clinical routine as the industrial production of penicillin started in 1942.^15^ Around the same time researchers at IOC began using penicillin manufactured in the Institute to treat some bacterial infections.^16^ Meanwhile other researchers continued traveling to the front where “progress was being carved out”. Their reports, published in the institutional journal *Memórias do Instituto Oswaldo Cruz*, describe the attempts to control the “textbook” parasites and their vectors that were hampering the workers progress on the building of roads and essential infrastructure. The state of abandonment of the population in the inner regions of Brazil was astonishing by any metrics or perspective. This could be summarised through the Cesar Pinto words, the researcher who in 1944 commented about the clinical condition of a young patient simultaneously infected by several parasites: “*...he is an authentic treatise on the pathology of parasitic diseases, as he was found to be infested by the following etiological agents: Plasmodium malariae, Plasmodium falciparum, Schistosoma mansoni, Necator americanus, Ascaris lumbricoides, Trichuris trichiura*.”^17^

The end of the 1930 decade was a time for the first national reports on diseases like leishmaniasis and respective vectors. That also meant the opportunity to engage young medical students and related professionals into a career pathway that would nurture the future generations of scientists for the country.^18^ ^,^ ^19^

By the end of this second quarter of a century the world had changed its scientific compass: it moved from Europe to the USA, which at the end of World War II had emerged as a military, economic, technological and scientific superpower. In his report to the president of USA in 1945, V. Bush, director of Office of scientific research and development, wrote:

“*Progress in the war against disease depends upon a flow of new scientific knowledge. New products, new industries, and more jobs require continuous additions to knowledge of the laws of nature, and the application of that knowledge to practical purposes*.”^20^

This report broadly defined an era in which governments took action to implement a national strategy of science and technology aiming to boost economic development. At least in theory, scientific investment would be translated into higher income and benefits for entire populations. In Brazil, the return to democracy (not without the usual political turmoil) meant that only at the end of the second quarter of a century two scientific agencies could emerge from the blueprints [Conselho Nacional de Desenvolvimento Científico e Tecnológico (CNPq) and Coordenação de Aperfeiçoamento de Pessoal de Nível Superior (CAPES), both created in 1951]. Nonetheless a long journey, full of bureaucracy and scarcity of basic resources, waited for the Brazilian researchers. For most of the following quarters of a century, we have been passengers in the science “*omnibus*”, anxious to get the driver seat but soon it was realised that being peripherically located, technologically lagged and science funding limited that seat was not yet available to the science enthusiasts from this land...

**Third quarter of a century**

“*The colonization efforts currently underway in various vast regions of Brazil have been a constant concern for scientific and governmental circles, to the point that a broad interdisciplinary project has recently been outlined for the prospective evaluation of the nosological potential of the areas influenced by the Trans-Amazonian Highway...*”

(João Carlos Pinto Dias, in Chagas disease survey in the Janaúba region, Minas Gerais, 1976)^21^

The political turmoil that followed the return to democracy in the second quarter of century would leave long-lasting changes in research organisations such as the IOC and the majority of universities in Brazil as well. The aftermath of that political dispute was a long military dictatorship (1964-1985) that redefined the pathway of every Brazilian organisation, including how the scientific endeavour should be planned and implemented as well as how the new generations of scientists should be trained. Under censorship the efforts of the scientific community were allowed to passively integrate a “grandiose” and authoritarian technocratic strategy to fully control Brazilian society, its movements and aspirations.

Meanwhile, progress in therapeutics for Chagas disease (Benznidazole and nifurtimox were already clinical trial),^22^ leishmaniasis (Pentavalent antimonials: meglumine antimoniate, sodium stibogluconate),^23^ malaria (the Chinese support to Vietnam war would leave us the artemisinin derivatives, though only in 1982 the West would know about it)^24^ ^,^ ^25^ and new chemicals to control the respective vectors expanded the possibilities to reduce the burden of the infectious diseases in populations living in the tropics. The goal of eliminating these diseases was in good prospect but in the final decades of the 20th century the social inequalities, economic crisis, natural complexity and survival strategy of microorganisms and their respective vectors returned this goal to the start point. Challenges including drug resistance, spread of vectors and microorganisms through human migration, and settlement in areas that should be under environmental protection demonstrated that quite a lot of basic research would be needed for disease control. Although an impressive knowledge about infectious diseases, their agents and vectors had been generated throughout the 20th century, it is clear now that we need much more to gain control over them. This third quarter of a century is characterised by a belief that as soon as new scientific evidence was published we could promptly translate it to a vaccine or effective drug and put it into clinical practice. It turned out to be a delusion and, in most cases, wishful thinking. Despite the ever increasing number of scientific articles, new methods, techniques, powerful instruments and public databases, frustration has accumulated. Leishmaniasis and Chagas disease are still treated with the same compounds developed in the 1940 and 1960 decades. In our land, things could not be worse: on top of the challenges naturally posed by elusive parasites and their vectors there were turbulent times of persecution and intolerance, when scientists were expelled from their laboratories and prohibited to do their research work...

**Fourth quarter of a century**

“*However, it is imperative that we are able to attenuate the social inequalities, to promote equal access to high quality education to all the population and not to the rich, and invest in science and technology, the greatest force that countries must have in order to face the world in the third millennium. The countries that have not yet been aware of this fact will be doomed.*”

(José Rodrigues Coura, at the 100th Anniversary of IOC)^26^

In the 1970 decade the field of biological sciences was reaching the plateau of a revolution that started twenty years early, when the structure of DNA was elucidated.^27^ In the next two decades the landscape of biomedical sciences would completely change after the announcement of a series of milestones like the unveiling of the genetic code,^28^ the discovery of reverse transcriptase,^29^ ^,^ ^30^ the DNA recombinant technology,^31^ the development of monoclonal antibodies,^32^ the discovery of gene interruptions^33^ and advances in nucleic acid sequencing methods.^34^ As a consequence of these breakthroughs big players were attracted to a science business game, and in collaboration with some of the researchers who got these achievements they shaped a new industry. Venture capitalists and ambitious entrepreneurs together gave rise to the gloomy and billionary industry of biotechnology. Later, the leaders of this new industry would forge alliances with members of another multibillionaire industry, the big pharmaceutical corporations, resulting in business models that brought new therapeutics and diagnostics products. This trend continued throughout the 1980 decade as a kind of preparation for the “omics” revolution that was coming out at the end of the 20th century.

In plain contrast to this wide biomedical revolution and expansion to the business arena, the IOC was struggling to keep its prestige and to rebuild trust and influence. Fifteen years after the beginning of the military dictatorship in Brazil, things were not scoring better in terms of strategy and purpose for the IOC. During an informal conversation with this author, Professor José Rodrigues Coura, the researcher who accepted the job of leading this institute at the end of 1970 decade, said: “*People at IOC in those years were not enthusiastic about their role as investigators, salaries were not attractive, research work was not strategically oriented, and even the Institute journal that was created 70 years earlier was on the verge of extinction.*”. In fact, between 1977 and 1979 Memórias do IOC did not publish any article. Prof. Coura reorganised the Institute and brought the Memórias do IOC back to the publishing arena.

The “rebirth” of IOC was in alignment with the prospect that arose when a new and big organisation was created by the military dictatorship: the Fundação Oswaldo Cruz, Fiocruz for short. Officially created in May 1970 to take the leading role of public health research organisation under the ministry of health, Fiocruz encompassed several isolated research organisations among which the IOC, as defined by the Federal Decree n. 66,624 May 22th 1970:

“Art 1. The Human Resources for Health Foundation is hereby transformed into the Fundação Instituto Oswaldo Cruz, and the Instituto Oswaldo Cruz and the Prophylactic Products Service of the National Department of Rural Endemics of the Ministry of Health are incorporated into it.”

(Available from: https://www2.camara.leg.br/legin/fed/decret/1970-1979/decreto-66624-22-maio-1970-408087-publicacaooriginal-1-pe.html).

Gone were the days of an organisation with “relative autonomy” to decide its own path and strategy. As part of a big conglomerate (Fundação Oswaldo Cruz) the IOC had from that moment on to share history and to dispute resources with several other research organisations towards a common goal: research and development of products/services/innovations for Brazilian public health. Such a goal is not different from that one in the “good old days” but now it is not alone in the long and winding road of research in human infectious diseases and related public health challenges.

**Fifth quarter of a century**

“*...about the first issue of Memórias is not only a ‘looking back’ to 20th century challenges in tropical medicine but an opportunity to reflect on what lessons have we learnt and the mistakes we should not repeat 115 years later.*”

(Editorial of Memórias do IOC journal at its 115th Anniversary)^35^

Success is expected from every organisation, and to help compose the winning narrative there are the “symbolic” achievements (or in reverse mode narrative, the absence of failures). That drives us to one relevant question in the ‘five quarter of a century’ trajectory of the IOC: among the many failures this organisation has undergone (yes, failures are worth remembering too) which one should not be allowed to happen? Perhaps the answer lies more on a personal choice rather than a rigorous analysis but certainly the most symbolic one was not finishing what Carlos Chagas started in 1908: to expand the knowledge about *Trypanosoma cruzi* by breaking the boundaries of a symbolic frontier: the genome composition of this protozoan parasite. At the end of 20th century genomics science (or “the omics” as we may call it today) was still taking its first steps. The sequencing technology was not yet massive, *i. e.*, the Sanger method was performed on semi-automated machines as the standard choice for revealing genome sequences. In 1994 researchers from IOC took part in a Latin American network to sequence the genomes of *T. cruzi* and *Leishmania braziliensis*. However, the ultimate goal (the complete genome sequence) was not attained by this initiative. At the end of the century, when genomics science gained momentum, the leadership of the IOC did not make the move to align or re-orient its strategic goals towards the achievement of that historical milestone. In that crucial moment the institutional narrative for the next generation should have been “*habemus T. cruzi codicem geneticum*”, widening the road opened by Carlos Chagas nine decades earlier. Instead, we could only register that in 2005 the *T. cruzi* genome sequence was finally published by a consortium of USA and European researchers (with the collaboration of some Brazilian researchers).^36^ The symbolism of this historical “achievement” was lost, but certainly there will be other ones, and here remains the desire that the next time a better alignment in leadership, strategic goals and institutional perspective might happen. A report on the objectives of this Latin American network to get *T. cruzi* genome sequenced can be read here.^37^

As we expect that many more “quarters of a century” be celebrated in the future, and not forgetting the risk of “falling and failing under the harsh reality of any organisational culture”, it comes the time to think of “recommendations” for this Institute to continue paving the current roads while opening new ones for innovations and problem solutions. From quite a long list that this author alone is unable to declare, two relevant ones should deserve a brief mention:

(i) *to embrace the risk of failures in every institutional activity and learn from them as they happen*. This is especially relevant for the set of activities involving the “graduate training programs” (the main source of underpaid workforce for knowledge generation in this Institute and other research organisations in Brazil). They are currently grounded on the concept that all of the research projects developed by D.Sc. candidates and their supervisors (it is also valid for post doc projects) can *never*, *absolutely never fail* and must be completed in a rigid time frame. Such a practice is a recipe to keep getting more of the same things, side moving about consolidated knowledge, and surely contributing to maintain a “safe” distance from the bold, risky, very innovative and disruptive ideas and far away from revealing the next breakthrough or solution.

(ii) *to build an Institute of an agile decision making process, oriented by a focused strategic planning, that promotes and develops an organisational culture engaged to “make more from scarce resources*” as well as committed to reduce the conflicts of interest between individual incentives (academic freedom to choose what is more personally rewarding) and the institutional priorities. If a strategy needs a re-orientation or it is failing to reach the planned goals we do not need to go on through lengthy and sterile discussions about what to do next. Organisations that look constantly for consensus about strategic goals, operational routines and for every granular aspect of its daily life are doomed to a slow pace and will lag behind the major advances that unfold around its core activity (not to mention that they fall within the big risk of losing influence, shutting down operations and closing the doors).

The series of events at the end of the first quarter of the 21st century seems to be merciless with organisations that do not adapt or evolve to get *pari passu* with this fast rate of change. There shall be hopes for the flourishing of both effective change and efforts that will assure Instituto Oswaldo Cruz many more vibrant *quarters of a century,* plenty of *Rerum Novarum* and disruptive solutions to Brazilian public health challenges.

## Data Availability

The contents underlying the research text are included in the manuscript.
